# proBDNF expression induces apoptosis and inhibits synaptic regeneration by regulating the RhoA-JNK pathway in an in vitro post-stroke depression model

**DOI:** 10.1038/s41398-021-01667-2

**Published:** 2021-11-10

**Authors:** Bangkun Yang, Lesheng Wang, Ying Nie, Wei Wei, Wenping Xiong

**Affiliations:** 1 Department of Neurosurgery, Zhongnan Hospital of Wuhan University, Wuhan, Hubei P. R. China; 2 Department of Pediatrics, Wuhan No.1 Hospital, Wuhan, Hubei P. R. China

**Keywords:** Depression, Neuroscience

## Abstract

Brain-derived neurotrophic factor (BDNF) plays an important role in the pathophysiology of post-stroke depression (PSD). However, the precise function and potential mechanism of proBDNF, the precursor form of BDNF, are unknown. In our study, a PSD-like model was established by treating neuronal cells with oxygen-glucose deprivation and corticosterone. We found that the protein proBDNF levels were significantly higher in the cortex and hippocampus in the PSD group than in the control group, suggesting that proBDNF plays a role in the pathophysiology of PSD. Furthermore, we re-established the PSD-like cell model using recombinant p75 neurotrophin receptor (p75NTR) or silencing c-Jun N-terminal kinase (JNK), and found that the PSD-induced upregulation of proBDNF was inhibited by recombinant p75NTR and JNK silencing (siJNK), and increased cellular apoptosis. Moreover, the application of recombinant p75NTR and siJNK in the PSD-like cell model significantly reversed the expression of apoptosis-related and depression-related proteins and decreased cellular apoptosis. Our findings suggest that proBDNF is involved in neural plasticity in PSD in vitro. The RhoA-JNK signaling pathway is activated after proBDNF binds to the p75NTR receptor, followed by the expression of apoptosis-related proteins (PSD95, synaptophysin, and P-cofilin), which contribute to PSD progression. The mechanism might involve the promotion of cellular apoptosis and the inhibition of nerve synapses regeneration by proBDNF.

## Introduction

Post-stroke depression (PSD) refers to a common neuropsychiatric complication characterized by low mood, loss of interest, and cognitive dysfunction, in addition to symptoms of neurological impairment after stroke [1–3]. Recent studies have reported the prevalence of PSD between 5 and 63%. PSD hinders the recovery of neurological function and the ability of daily living in stroke patients, and is closely related to the social avoidance and high mortality of stroke patients [4, 5]. Stroke is the most direct cause of PSD; however, the specific mechanisms of PSD are unknown [6]. Although several theories have been proposed, including the psychological stress theory, neurotransmitter theory, and inflammatory factor theory, these theories mainly focused on the patients’ clinical manifestations and related factors. Mature animal or cell models of PSD and basic research regarding PSD pathogenesis are lacking.

The brain-derived neurotropic factor (BDNF) is one of the most widely distributed and abundant members of the nerve growth factor family in the mammalian brain [7, 8] and is closely related to the occurrence and development of many neurological diseases, especially PSD [8, 9]. BDNF is key in understanding PSD and the complex interactions between antidepressants and post-stroke recovery [10, 11]. BDNF can protect against ischemic brain injury and reduce neuronal apoptosis after glucose deprivation [12]. BDNF signal transduction plays an important role in hippocampal neurogenesis and promotes functional recovery after stroke in the perimeningeal cortex [13]. The BDNF Val66Met polymorphism can alter the association between stroke and depression [14].

There are three forms of BDNF in the central nervous system: its precursor proBDNF, mature BDNF (mature form), and BDNF pro-peptide [15]. The expressions of proBDNF and mature BDNF show dynamic changes at different stages of brain development [16]. Research has revealed that proBDNF and mature BDNF have similar biological activities. As a functional protein, proBDNF binds to the low-affinity nerve growth factor receptor p75 (p75NTR) to further activate downstream signals, such as tumor necrosis factor-related receptor 6 and Ras homolog gene family member A (RhoA), to induce apoptosis [17]. In our previous publication, we found that in a depression model of learned helplessness rats, the expression of proBDNF was significantly increased in the prefrontal cortex and hippocampus and significantly decreased in the nucleus accumbens. proBDNF may play a different role than BDNF [18]. RhoA has multiple targets, including c-Jun and N-terminal kinase (JNK) [19, 20]. The JNK signaling pathway is related to neurodegeneration, ischemia-reperfusion, and other diseases [21]. However, the precise function and potential mechanisms of proBDNF in PSD are unknown. Here, we propose that the RhoA-JNK signal pathway is activated by proBDNF, which promotes cellular apoptosis and inhibits nerve synapse regeneration.

## Materials and methods

### Cell culture

Cortical and hippocampal neurons derived from embryonic day 15 (E15) C57Bl6/J mice were dissociated in 0.25% trypsin (Sigma, St Louis, MO, USA) at 37 °C for 10 min and seeded in a 6-well plate (1.0 × 10^5^ per well). Neurons were maintained in 2 mL neurobasal medium containing B27 supplement (2%; Invitrogen, Carlsbad, CA, USA), penicillin-streptomycin (50 μg/mL penicillin, 50 U/mL streptomycin; Invitrogen), and L-glutamine (2 mM; Invitrogen) at 37 °C and 5% CO_2_ for 7 days in vitro, with half the medium being replaced with the fresh warm medium on day 3 and day 6. All experimental protocols were approved by the Wuhan University Institutional Animal Care and Use Committee in accordance with the China Code of Practice for the Care and Use of Animals for Scientific Purposes.

### Production of recombinant p75NTR and silencing of JNK

The human p75NTRcDNA sequence was amplified by PCR using primers 5′-GCGGATCCAAAGGAGGCATGCCCC-3′, and 5′-CGCGGATCAGTGGTGGT GGTGGTGGTGAATCCAA-CGGCCAGGGAT-3′, and cloned into the pVTBac vector (D. Tessier, CNRC, Montreal, Canada). The pVTBac plasmid encodes the melittin signal peptide, which allows efficient secretion of p75NTR. To produce p75NTR–His6, cortical and hippocampal neuronal cells were infected with the recombinant baculoviruses in a serum-free medium (Invitrogen). At 3 days post-infection, the medium was collected and clarified by centrifugation at 3000 g for 15 min. The supernatant was extensively dialyzed against 50 mM Tris buffer (pH 8) at 4 °C and then incubated overnight with Ni-NTA resin (Qiagen, Agilent Technologies, Inc.). The resin was washed once with 20 mM imidazole in 300 mM NaCl, 10 mM Tris (pH 8) buffer; bound proteins were eluted with 100 mM imidazole in the same buffer. The p75NTR-containing fractions were pooled, concentrated, and then dialyzed against PBS.

### Cell transfection

Cortical and hippocampal neuronal cells were seeded in 6‑well plates at density of 4 × 10^5^ cells per well. After 24 h, the C57Bl6/J neuronal cells were transfected with JNK1 small interfering RNA (siRNa; Sigma, St. Louis, MO, USA) using Lipofectamine 2000 reagent (Invitrogen). The sequences of the negative control siRNA and the JNK1 siRNAs are shown as follows:

negative control siRNA: 5′‑CCAGGACGAACAGATGTTT‑3′;

JNK1 siRNA‑374: 5′‑GCTCATGGATGCAAATCTT‑3′;

JNK1 siRNA‑647: 5′‑GGGCTACAAGGAGAACGTT‑3′;

JNK1 siRNA-919: 5′‑CCAGTCAGGCAAGAGATTT‑3′.

### Experimental groups

Primary neurons were treated with oxygen and glucose deprivation (OGD) for 2 h (the OGD group was added to a serum-free culture medium at 37 °C for 24 h and then placed in a closed hypoxia chamber at 37 °C for 8 h), followed by the addition of 500 μmol/L corticosterone (when grown to 60–70% confluence, the cells were treated with corticosterone for 36 h) to continue the culture process using the method previously described [22–26]. Recombinant p75NTR or JNK siRNA was added into neuronal cells in different groups.

We used six subgroups of prefrontal cortical neurons and hippocampal neurons, respectively. The prefrontal cortex and hippocampal neuron groups from 15-day gestational mice were divided into six groups: (i) control group: primary neurons; (ii) stroke-like group: primary neurons treated with OGD for 2 h; (iii) depression-like group: primary neurons treated with corticosterone; (iv) PSD-like group: primary neurons treated with corticosterone after OGD treatment; (v) PSD-like + recombinant p75NTR group: recombinant p75NTR was transfected into primary neurons after corticosterone and OGD treatment; and (vi) PSD-like + siJNK group: siJNK was transfected into primary neurons after corticosterone and OGD treatment. The different treatments were sequentially added according to the above-mentioned order. Detailed grouping information is summarized in Table 1.

**Table 1:** Grouping of experimental cells and outline of the experiments.

Prefrontal cortex neurons		Hippocampal neurons	
A1	Control group	A2	Control group
B1	Stroke-like group	B2	Stroke-like group
C1	Depression-like group	C2	Depression-like group
D1	PSD-like group	D2	PSD-like group
E1	PSD-like+p75NTR group	E2	PSD-like+p75NTR group
F1	PSD-like+siJNK1 group	F2	PSD-like+siJNK1 group

### Enzyme-linked immunosorbent assay quantification of proBDNF expression in neuronal cells

Quantification of proBDNF was assessed using the Biosensis proBDNF RapidTM enzyme-linked immunosorbent assay (ELISA) Kit (Biosensis Pty Ltd. West Thebarton, SA, Australia). Briefly, 100 µL of diluted proBDNF samples were added to the pre-coated 96-well microplates. One hundred microliters of detection antibody were added to each well. After shaking and washing, 100 µL of the 1 × streptavidin-HRP was added to each well, then 100 µL of tetramethylbenzidine was added into each well, and the plate was incubated at room temperature for 10–15 min. The reaction was stopped by adding 100 µL of the stop solution to each well. The absorbance at 450 nm was read using a plate reader. Experiments were conducted in triplicate and repeated at least three times.

### Transmission electron microscopy of hippocampal and prefrontal dendritic spines

Electron microscopic examination was performed on material from C57BL/6 mice. Immunoperoxidase labeling was first examined to identify the types of cellular processes (dendrites, axons, dendritic spines, axon terminals, and glial cells) that were immunoreactive to the EphA4 and EphB2 antibodies. Six photographs per region were randomly taken at 2900×, 6800×, and 13,000× magnification covering 300 μm^2^, and were used to assess the relative proportions of each type of process that was labeled.

### Detection of cellular apoptosis via flow cytometry

Apoptotic cells were detected using an Annexin V-FITC Apoptosis Detection kit (Beyotime Institute of Biotechnology). Briefly, cells were collected by centrifugation at 1000 g for 5 min at room temperature. Cells were stained with 5 µL annexin V-FITC and 10 µL propidium iodide (PI) and incubated at room temperature in the dark for 15 min. Subsequently, the apoptotic cells were immediately detected using a NovoCyte flow cytometer (ACEA Bioscience, Inc.) and analyzed using NovoExpress software (version 1.2.5; ACEA Bioscience, Inc.). The apoptotic rate of cells was calculated by adding the percentage of early apoptotic (Annexin V-positive and PI-negative) cells and late apoptotic (Annexin V-positive and PI-positive) cells.

### Cell viability detected by CCK-8 assay

Log phase cells were trypsinized and seeded into a 96-well plate, with 10,000 cells/well. The plate was incubated for 24 h at 37 °C in CO_2_ (5%). The medium of each group was replaced with a medium containing a specific concentration of drug corresponding to each of the 100-μL cell samples, and the control group medium was replaced with a medium containing a solvent. Ten microliters of CCK-8 were added into each well, and the plate was incubated for 2 h. Absorbance at 450 nm was measured using a microplate reader.

### Quantitative reverse transcription PCR(RT-qPCR) assay to detect mRNA levels of p75NTR, JNK1, and RhoA in all groups

Total RNA was extracted from cells using the TRIpure Total RNA Extraction Reagent (ELK Biotech Co., Ltd.) and reverse-transcribed into cDNA using oligo (dT)15, random primers (ELK Biotech Co., Ltd.), M‑MLV reverse transcriptase (ELK Biotech Co., Ltd.), dNTPs, 5X buffer, and RNase inhibitor (ELK Biotech Co., Ltd.) at 25 °C for 10 min, 42 °C for 50 min, and 80 °C for 10 min. RT-qPCR was subsequently performed to analyze the expression levels of p75NTR, RhoA, and JNK1 using cDNA samples, primers (ELK Biotech Co., Ltd.), SYBR Green (ELK Biotech Co., Ltd.), and 2X Taq PCR MasterMix (ELK Biotech Co., Ltd.). The following thermocycling conditions were used for the qPCR: initial denaturation at 94 °C for 3 min; followed by 40 cycles at 95 °C for 10 s, 58 °C for 10 s and 72 °C for 30 s; and a final extension at 72 °C for 150 s. The sequences of primers used for qPCR are shown in Table 2. The 2^–ΔΔCq^ method was used to quantify the relative expression levels of p75NTR, RhoA, and JNK1 [27]. GAPDH was used as a loading control.

**Table 2:** Sequences of the primers used in RT-PCR.

Name	Version	Base sequence (5′-3′)		Tm	CG%
GAPDH	NM_008084.3	F	TGAAGGGTGGAGCCAAAAG	58.3	52.6
		R	AGTCTTCTGGGTGGCAGTGAT	58.4	52.4
RhoA	NM_001313961.1	F	GGAACAAGAAGGACCTTCGG	58.6	55
		R	CCACGTCTAGCTTGCAGAGC	58.5	60
JNK1	NM_001310452.1	F	CAGCACCCATACATCAACGTC	58.6	52.4
		R	GCCCTCTTATGACTCCATTCTTAG	58.8	45.8
p75NTR	NM_033217.3	F	ACCTCATTCCTGTCTATTGCTCC	59.3	47.8
		R	GTGTGGGTCTGCTGGTCGT	58.3	63.2

### Western blotting

All neurons were lysed in a 2 × Laemmli sample buffer. The samples were then sonicated and loaded onto a 12% sodium dodecyl sulfate-polyacrylamide gel. Protein was transferred to a polyvinylidene fluoride (PVDF) membrane. The membrane was incubated overnight with 5% nonfat milk in Tris-buffered saline containing 0.05% Tween 20 (TBS-T). Membranes were incubated with primary antibody (mouse anti-proBDNF [1lg/mL; Invitrogen] and rabbit antitubulin [1:5 000; Cell Signaling, Danvers, MA, USA]) overnight at 4 °C. After washing, the membranes were incubated with horseradish peroxidase-labeled secondary antibody for 1 h. The secondary antibodies used for detection were ECL antimouse IgG (Amersham, Piscataway, NJ, USA) and ECL antirabbit IgG (Amersham). Signals were visualized using chemiluminescence (Perkin Elmer, Waltham, MA, USA).

### Statistical analysis

All quantitative data were analyzed using SPSS 25.0 software (IBM Corp., Armonk, New York, USA) and presented as mean ± standard error of mean (SEM). All data were analyzed using one-way analysis of variance (ANOVA), except for the standard penetration test data, which were analyzed using the repeated measures test. *P* values <0.05 were considered significant.

## Results

### proBDNF is upregulated in the neuronal cells following PSD

**Fig. 1:**
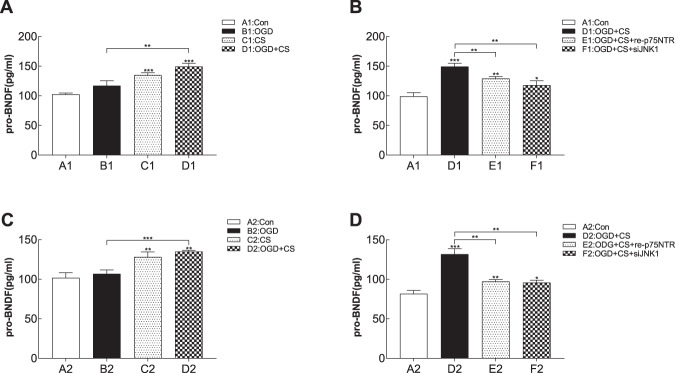
Enzyme-linked immunosorbent assay (ELISA) of proBDNF in prefrontal cortical and hippocampal neurons (*n* = 3 per subgroup). **A**, **B** Level of proBDNF in prefrontal cortical neurons with different treatments. **C, D** Level of in hippocampal neurons with different treatments. proBDNF content is expressed in pg/mL. Error bar represents mean ± SEM. *P < 0.05, **P < 0.01, ***P < 0.001, ****P < 0.0001 versus control group. Con control, OGD oxygen-glucose deprivation, CS corticosterone.

ELISA was performed to detect proBDNF in different groups. Results of the one-way ANOVA analysis of proBDNF data are as follows: antFront: F_(3,8)_ = 38.76, *p* < 0.0001; *p*_(A1 vs. B1)_ = 0.05, *p*_(A1 vs. C1)_ < 0.001, *p*_(A1 vs. D1)_ < 0.001, *p*_(B1 vs. D1)_ < 0.01 (Fig. 1A); Hippo: F_(3,8)_ = 26.91, *p* = 0.001; *p*_(A2 vs. B2)_ = 0.35, *p*_(A2 vs. C2)_ < 0.01, *p*_(A2 vs. D2)_ < 0.01, *p*_(B2 vs. D2)_ < 0.001 (Fig. 1B). In the depression-like and PSD-like groups, proBDNF in both types of neurons was upregulated. Meanwhile, proBDNF levels were lower in the PSD-like group than in the stroke-like and depression-like groups. In contrast, proBDNF levels in the PSD-like plus recombinant p75NTR and PSD-like plus siJNK groups were significantly lower than those in the PSD-like group (antFront: F_(3,8)_ = 28.12, *p* < 0.001; *p*_(A1 vs. D1)_ < 0.001, *p*_(A1 vs. E1)_ < 0.01, *p*_(A1 vs. F1)_ = 0.03, *p*_(D1 vs. E1)_ < 0.01; *p*_(D1 vs. F1)_ < 0.01, Fig. 1C), (Hippo: F_(3,8)_ = 62.2, *p* < 0.0001; *p*_(A2 vs. D2)_ < 0.001, *p*_(A2 vs. E2)_ < 0.01, *p*_(A2 vs. F2)_ = 0.01, *p*_(D2 vs. E2)_ = 0.001; *p*_(D2 vs. F2)_ < 0.01, Fig. 1D). These data indicate that p75NTR and JNK are involved in the process of increasing proBDNF induced in the PSD-like model.

### PSD-like model treatments resulted in significant decreases in cell viability

**Fig. 2:**
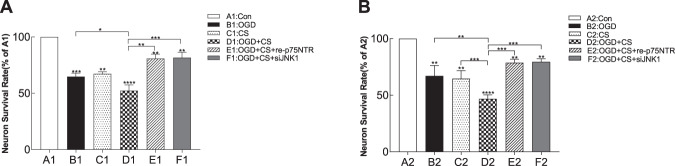
Neuron survival rate using CCK-8 assay in six groups. (*n* = 3 per group) 1, Prefrontal cortical neurons. 2, Hippocampal neurons. **A** Neuron survival rate of the prefrontal cortex. **B** Neuronal survival rate of hippocampal neurons. Data are expressed as a percentage of the control group. Data are presented as mean (columns) and SD (error bar). “*” on the top of each column represents the *P* value compared with the control group (Group A). *P < 0.05, **P < 0.01, ***P < 0.001, ****P < 0.0001. Con control, OGD oxygen-glucose deprivation, CS corticosterone.

We evaluated whether the PSD-like model caused a higher neuron death rate, by using the CCK-8 assay to test neuronal survival rates. Results of the one-way ANOVA analysis of cell viability are as follows: antFront: F_(5,24)_ = 95.88, *p* < 0.0001; *p*_(B1 vs. D1)_ = 0.02, *p*_(D1 vs. E1)_ < 0.001, *p*_(D1 vs. F1)_ < 0.0001 (Fig. 2A); Hippo: F_(5,24)_ = 41.09, *p* < 0.0001; *p*_(B2 vs D2)_ < 0.001, *p*_(D2 vs E2)_ < 0.0001, *p*_(D2 vs F2)_ < 0.001 (Fig. 2B). Cell viability was lower in the PSD-like group than in the other groups (antFront: *p*_(A1 vs. D1)_ < 0.0001; Hippo: *p*_(A2 vs. D2)_ < 0.0001). There was a distinct reduction in the neuronal survival rate of both hippocampal and prefrontal cortical neurons in the PSD-like group. We explored whether p75NTR and the JNK pathway play a role in neuronal viability. Neuron survival rate was significantly increased by inhibition of these two receptors by competitive recombinant p75NTR and siRNA in PSD-like models. These findings confirmed that p75NTR and the JNK pathway play important roles in the induction of neuronal death in PSD-like models.

### PSD can result from or lead to apoptosis

**Fig. 3:**
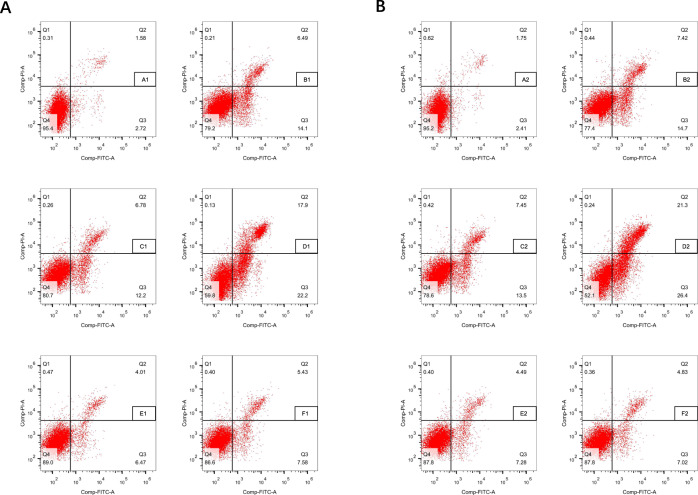
AnnexinV-FITC flow cytometry to detect the rate of apoptosis in the prefrontal cortex and hippocampal neurons derived from listed subgroups in Table 1. The rate of apoptosis is marked in the right upper quadrant. **A** Flow cytometry of prefrontal cortex neurons. **B** Flow cytometry of hippocampal neurons.

The stroke-like, depression-like, and PSD-like groups all presented with higher apoptotic rates than the control group. Neurons in the PSD-like group showed the highest apoptotic rate. The apoptotic rate was 21.3% in the PSD-like group vs. 1.75% in the control group for hippocampal neurons and 17.9 vs. 1.58% for prefrontal cortical neurons. Furthermore, the apoptotic rate was deceased when the JNK1 pathway and p75NTR were inhibited in both neuron subgroups (Fig. 3). Flow cytometry analysis indicated that the PSD-like model dramatically aggravated apoptosis of hippocampal neurons and prefrontal cortical neuronal cells; however, proBDNF-triggered apoptosis was attenuated after co-transfection with a JNK1 or p75NTR inhibitor.

### The number of synapses with a mature morphology were reduced

**Fig. 4:**
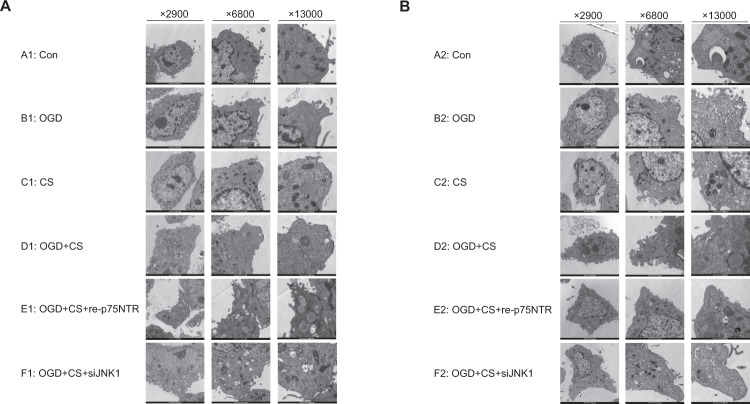
Electron microscopy of hippocampal and prefrontal cortical neurons (Magnification ×2900, ×6800, ×13,000). **A** Electron microscopy of hippocampal neurons. **B** Electron microscopy of prefrontal cortical neurons. Dendrite counts: A1-14, B1-10, C1-8, D1-3, E1-7, F1-9; A2-16, B2-14, C2-10, D2-2, E2-7, F2-6. Con control, OGD oxygen-glucose deprivation, CS corticosterone. A1: Con; B1: OGD; C1: CS; D1: OGD+CS; E1: OGD+CS+re-p75NTR; F1: OGD+CS+siJNK1; A2: Con; B2: OGD; C2: CS; D2: OGD+CS; E2: OGD+CS+re-p75NTR; F2: OGD+CS+siJNK1.

To study the role of proBDNF on dendritic spines, we counted the number of dendritic spines in hippocampal and prefrontal neurons. PSD caused marked pathological changes by decreasing dendrite counts in hippocampal neurons and prefrontal cortical neurons (Fig. 4). In addition, neurons in the stroke group, depression group, and PSD group all showed lower dendrite counts than neurons in the control group, as observed by electron microscopy (counts: A1-14, B1-10, C1-8, D1-3; A2-16, B2-14, C2-10, D2-2). Dendrite counts of neurons in the PSD group were lower than those in the stroke group and depression group, suggesting that the impairment of synaptic and neuron morphological characteristics is more severe in the PSD group than in the simple stroke or depression groups. Inhibition of p75NTR and JNK1 partially reversed the morphological impairment (counts: E1-7, F1-9; E2-7, F2-6).

### The proBDNF/p75NTR/RhoA/JNK signaling pathway was activated in the PSD-like model

**Fig. 5:**
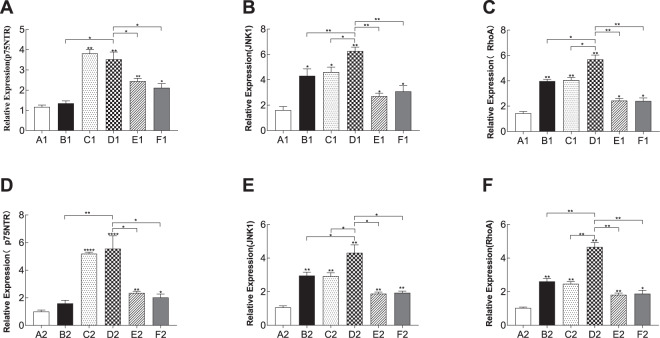
Reverse transcriptase quantitative PCR (RT-qPCR) assay to detect the mRNA levels of p75NTR, JNK1 and RhoA in all groups (*n* = 3 per subgroup). **A** p75NTR mRNA level of prefrontal cortical neurons. **B** JNK1 mRNA level of prefrontal cortical neurons. **C** RhoA mRNA level of prefrontal cortical neurons. **D** p75NTR mRNA level of hippocampal neurons. **E** JNK1 mRNA level of hippocampal neurons. (F) RhoA mRNA level of hippocampal neurons. Con control, OGD oxygen-glucose deprivation, CS corticosterone. A1: Con; B1: OGD; C1: CS; D1: OGD+CS; E1: OGD+CS+re-p75NTR; F1: OGD+CS+siJNK1; A2: Con; B2: OGD; C2: CS; D2: OGD+CS; E2: OGD+CS+re-p75NTR; F2: OGD+CS+siJNK1.

**Fig. 6:**
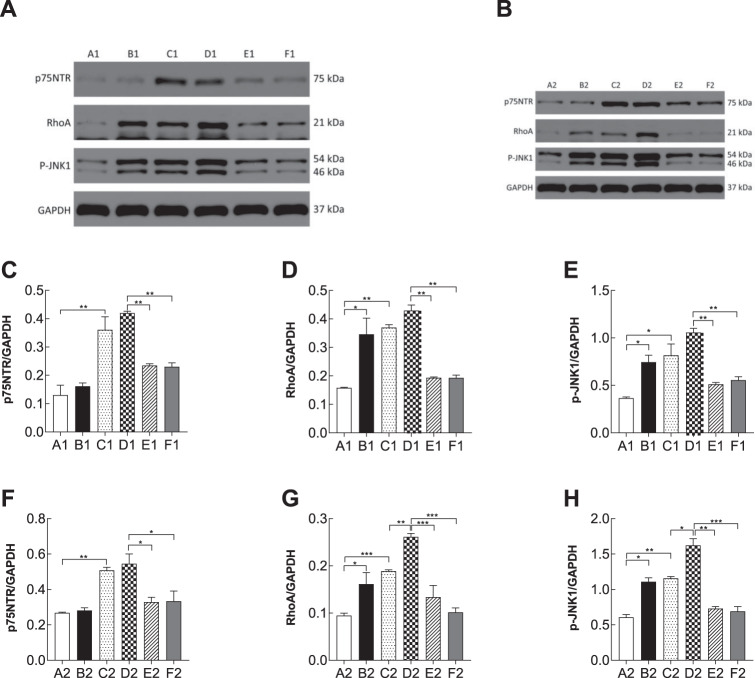
Representative western blot analysis and quantification of densitometries of western blot band in all groups (*n* = 3 per subgroup). (**A** and **B**) p75NTR, RhoA, and JNK1/GAPGH expression levels in neuronal cells were detected using western blotting. (**C**–**E**) Bar chart showing the ratio of p75NTR, RhoA, and JNK1 protein to GADPH in prefrontal cortical neurons. **F**–**H** Bar chart showing the ratio of p75NTR, RhoA, and JNK1 protein to GADPH in hippocampal neurons.Con control, OGD oxygen-glucose deprivation, CS corticosterone. A1: Con; B1: OGD; C1: CS; D1: OGD+CS; E1: OGD+CS+re-p75NTR; F1: OGD+CS+siJNK1; A2: Con; B2: OGD; C2: CS; D2: OGD+CS; E2: OGD+CS+re-p75NTR; F2: OGD+CS+siJNK1.

proBDNF that preferentially binds to its co-receptor p75NTR plays an important role in neuronal apoptosis. In prefrontal cortical neurons, the mRNA levels of p75NTR (3.523 ± 0.213 in the PSD-like group vs. 1.348 ± 0.187 in the stroke group, *n* = 3, *p* = 0.01; vs. 2.443 ± 0.227 in the PSD-like+re-p75NTR group, *n* = 3, *p* = 0.04; vs. 2.116 ± 0.239 in the PSD-like+siJNK1 group, *n* = 3, *p* = 0.03, Fig. 5A), RhoA (5.687 ± 0.158 in the PSD-like group vs. 3.975 ± 0.142 in the stroke group, *n* = 3, *p* < 0.01; vs. 4.064 ± 0.172 in the depression-like group, *n* = 3, *p* = 0.03; vs. 2.438 ± 0.185 in the PSD-like+re-p75NTR group, *n* = 3, *p* < 0.01; vs. 2.4 ± 0.211 in the PSD-like+siJNK1 group, *n* = 3, *p* < 0.01, Fig. 5B), and JNK (6.273 ± 0.363 in the PSD-like group vs. 4.315 ± 0.275 in the stroke group, *n* = 3, *p* < 0.01; vs. 4.616 ± 0.282 in the CS group, *n* = 3, *p* = 0.02; vs. 2.709 ± 0.218 in the PSD-like+re-p75NTR group, *n* = 3, *p* < 0.01; vs. 3.092 ± 0.309 in the OGD+CS+siJNK1 group, *n* = 3, *p* < 0.01, Fig. 5C) were elevated in the PSD-like group. Similarly, the mRNA levels of p75NTR (5.562 ± 0.539 in the PSD group vs. 1.595 ± 0.192 in the stroke group, *n* = 3, *p* = 0.01; vs. 2.346 ± 0.53 in the PSD-like+re-p75NTR group, *n* = 3, *p* = 0.02; vs. 2.026 ± 0.514 in the PSD-like+siJNK1 group, *n* = 3, *p* = 0.01, Fig. 5D), RhoA (4.622 ± 0.254 in the PSD group vs. 2.611 ± 0.184 in the OGD group, *n* = 3, *p* < 0.01; vs. 2.466 ± 0.172 in the CS group, *n* = 3, *p* < 0.01; vs. 1.82 ± 0.164 in the PSD-like+p75NTR group, *n* = 3, *p* < 0.01; vs. 1.878 ± 0.196 in the PSD-like+siJNK1 group, *n* = 3, *p* < 0.01, Fig. 5E), and JNK (4.316 ± 0.396 in the PSD-like group vs. 2.961 ± 0.299 in the OGD group, *n* = 3, *p* = 0.02; vs. 2.926 ± 0.299 in the CS group, *n* = 3, *p* = 0.02; vs. 1.884 ± 0.28 in the PSD-like+p75NTR group, *n* = 3, *p* = 0.01; vs. 1.937 ± 0.28 in the PSD-like+siJNK1 group, *n* = 3, *p* < 0.01, Fig. 5F) in hippocampal neurons were elevated in the PSD-like group. Moreover, the protein levels of p75NTR, RhoA, and JNK (Fig. 6) were significantly higher in the PSD-like group than in the other groups. These data indicate that proBDNF promotes PSD via the RhoA-JNK signaling pathway.

### Apoptosis-related proteins were induced by the RhoA-JNK signaling pathway

**Fig. 7:**
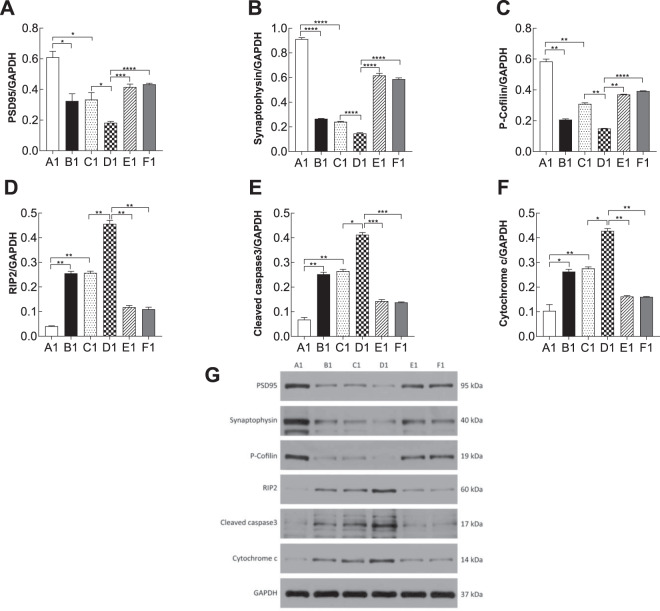
Statistical analysis of western blot profiles in prefrontal cortical neurons (*n* = 3). **A** PSD95. **B** Synaptophysin. **C** P-cofilin. **D** RIP2. **E** Cleaved caspase-3. **F** Cytochrome c. **G** PSD95, synaptophysin, P-cofilin, RIP2, cleaved caspase-3, cytochrome c /GAPGH expression levels in prefrontal cortical neuronal cells were detected using western blotting. Synaptic-related proteins and apoptosis-related proteins were upregulated in the stroke, depression and PSD groups, and knock down of p75NTR and JNK1 in PSD models reversed this effect. “*” on the top of each bar represents the P value compared to the control group. Error bars represent the mean ± SD. *P < 0.05, **P < 0.01, ***P < 0.001, ****P < 0.0001. Con control, OGD oxygen-glucose deprivation, CS corticosterone. A1: Con; B1: OGD; C1: CS; D1: OGD+CS; E1: OGD+CS+re-p75NTR; F1: OGD+CS+siJNK1; A2: Con; B2: OGD; C2: CS; D2: OGD+CS; E2: OGD+CS+re-p75NTR; F2: OGD+CS+siJNK1.

**Fig. 8:**
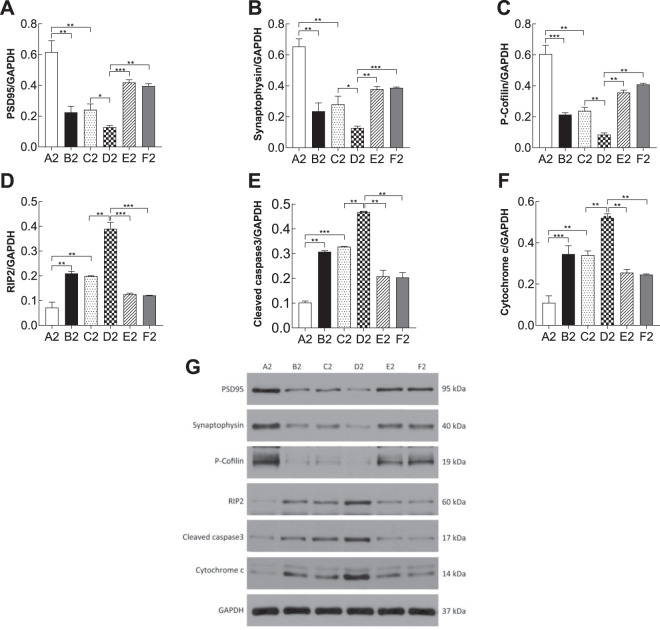
Statistical analysis of Western blot profiles in hippocampal neurons (*n* = 3). **A** PSD95. **B** Synaptophysin. **C** P-cofilin. **D** RIP2. **E** Cleaved caspase-3. **F** Cytochrome c. **G** PSD95, synaptophysin, P-cofilin, RIP2, cleaved caspase-3, cytochrome c /GAPGH expression levels in hippocampal neuronal cells were detected using western blotting. A similar effect was observed in hippocampal neurons to that observed in prefrontal cortical neurons. Error bars represent the mean ± SD. *P < 0.05, **P < 0.01, ***P < 0.001, ****P < 0.0001. Con control, OGD oxygen-glucose deprivation, CS corticosterone. A1: Con; B1: OGD; C1: CS; D1: OGD+CS; E1: OGD+CS+re-p75NTR; F1: OGD+CS+siJNK1; A2: Con; B2: OGD; C2: CS; D2: OGD+CS; E2: OGD+CS+re-p75NTR; F2: OGD+CS+siJNK1.

To investigate the molecular changes in different groups, western blotting was performed to analysis several biomarkers of synapsis and apoptosis. proBDNF contributes to PSD by regulating the expression of inflammatory factors, such as PSD95, synaptophysin, and P-cofilin, or by inhibiting the expression of antiapoptosis-related proteins including RIP2, cleaved caspase-3, and cytochrome c (Figs. 7 and 8). In the PSD models, there was a significant reduction in the synaptic-related proteins PSD-95(PSD-like group vs. depression-like group, *p1* = 0.03, *p2* = 0.04; vs. PSD-like+re-p75NTR group, *p1* < 0.01, *p*2 < 0.001; vs. PSD-like+siJNK1 group, *p1* < 0.01, *p2* < 0.01, Figs. 7A and 8A), synaptophysin (PSD-like group vs. depression-like group, *p1* < 0.001, *p*2 = 0.02; vs. PSD-like+re-p75NTR group, *p1* < 0.001, *p2* < 0.001; vs. PSD-like+siJNK1 group, *p1* < 0.001, *p2* < 0.01, Figs. 7B and 8B), and P-cofilin (PSD-like group vs. depression-like group, *p1* < 0.01, *p2* < 0.01; vs. PSD-like+re-p75NTR group, *p1* < 0.01, *p2* < 0.01; vs. PSD-like+siJNK1 group, *p1* < 0.01, *p2* < 0.0001, Figs. 7C and 8C). These data indicate the impairment of synaptic structures in PSD models. Impairment of synaptic structures was significantly reversed by inhibition of p75NTR and JNK1 suggesting that these two proteins play a role in PSD models. Moreover, the apoptosis-promoting proteins RIP2 (PSD-like group vs. depression-like group, *p1* < 0.01, *p2* < 0.01; vs. PSD-like+re-p75NTR group, *p*1 < 0.01, *p*2 < 0.001; vs. PSD-like+siJNK1 group, *p1* < 0.01, *p2* < 0.001, Figs. 7D and 8D), cleaved caspase-3 (PSD-like group vs. depression-like group, *p1* = 0.02, *p2* < 0.01; vs. PSD-like+re-p75NTR group, *p1* < 0.001, *p2* < 0.01; vs. PSD-like+siJNK1 group, *p1* < 0.001, *p2* < 0.01, Figs. 7E and 8E), and cytochrome c (PSD-like group vs. depression-like group, *p1* = 0.01, *p2* < 0.01; vs. PSD-like+re-p75NTR group, *p1* < 0.01, *p*2 < 0.01; vs. PSD-like+siJNK1 group, *p1* < 0.01, *p2* < 0.01, Figs. 7F and 8F) were distinctly upregulated in PSD models. These data indicate that inhibition of re-p75NTR and siJNK1 could significantly decrease the level of these three apoptotic markers.

## Discussion

The present study investigated the role of proBDNF in the regulation of PSD. There were two major findings: (i) proBDNF is involved in the pathophysiological process of PSD and (ii) proBDNF binds to the p75NTR receptor and activates RhoA/JNK signaling to promote cellular apoptosis and inhibit the regeneration of nerve synapses, thereby promoting the pathogenesis of PSD.

### ProBDNF plays a critical role in the OGD-CS-induced PSD model

The level of proBDNF in depression models is significantly increased in neuronal cells in vivo or in vitro [28, 29]. In our study, a PSD model was successfully induced by OGD combined with CS, generating PSD-like neuronal cells. As expected, our data suggest that proBDNF was increased in the depression and PSD groups, which is consistent with previous studies [30]. The level of proBDNF in primary neurons that were treated with corticosterone did not increase significantly; this finding is different from that of other studies [31]. The many anatomical differences between in vivo and in vitro experimental systems might explain this discrepancy. The level of proBDNF was significantly reduced in primary neurons treated with recombinant p75NTR and siJNK. siJNK can suppress the activation of JNK via RNA silencing [32]. The above findings might suggest that the upregulation of proBDNF is associated with the development and progression of PSD.

### proBDNF-mediated apoptosis in neuronal cells

Under physiological conditions, proBDNF induces neuronal apoptosis in vitro and in vivo after nerve injury [33, 34]. In the present study, our data indicated that the survival rate of prefrontal cortical or hippocampal neurons treated with recombinant p75NTR and siJNK was significantly higher than that in the PSD group. The rate of apoptosis in prefrontal cortical and hippocampal neurons treated with recombinant p75NTR and siJNK was significantly lower than that in the PSD group. These data indicate that p75NTR and JNK function as novel downstream factors in the PSD model. It has been reported that the JNK pathway is one of the signaling pathways linked to cellular apoptosis and the cell stress response [35, 36]. Zhang et al. studied the role of JNK in depression-like behavior induced by central lipopolysaccharide (LPS) infusion. They found that lipopolysaccharide infusion leads to depression-like behavior, accompanied by increased expression of pro-inflammatory cytokines and increased JNK activation [37]. p75NTR which is a member of the tumor necrosis factor receptor superfamily is a low-affinity receptor for neurotrophic factors. It is abundantly expressed during the early developmental stages of nerve cells, and induces cell proliferation, migration, differentiation, survival, apoptosis, synapse establishment, and nerve formation, and exerts multiple biological effects through different signal transduction pathways. A previous study showed that p75NTR might mitigate depression and prompt BDNF maturation [38], which may be counterproductive for PSD. Therefore, JNK and p75NTR are promising candidates as new therapeutic targets for PSD.

### The number of dendrites was reduced in the PSD model

The PSD model induced morphological changes in neuronal cells, particularly neuronal protrusions. To study the role of BDNF and proBDNF on the dendritic number, we counted the dendritic density of the prefrontal cortical and hippocampal neurons. The PSD group exhibited a significant reduction in dendritic density. Chronic unpredicted mild stress (CUMS)-induced depressive-like behaviors were accompanied by a significant reduction in spine density [39]. Hisatsugu et al. examined the effects of cleavage-resistant proBDNF on neuronal morphology using basal forebrain cholinergic neurons and hippocampal neurons. The study showed that proBDNF dramatically reduced the number of cholinergic fibers and hippocampal dendritic spines, without affecting the survival of these neurons [40].

### proBDNF promoted the expression of apoptosis-related proteins

Proteins associated with apoptosis can be classified as anti-apoptotic, proapoptotic, or BH3-only proteins [41]. In addition, cellular apoptosis is tightly controlled by apoptosis-associated proteins. To explore the potential mechanism underlying PSD, we studied the expression of apoptotic proteins related to RhoA, including PSD95, synaptophysin, P-cofilin, RIP2, cleaved caspase-3, and cytochrome c. RhoA and JNK are proapoptotic proteins that could promote the expression of apoptosis-related proteins. PSD95, synaptophysin, and P-cofilin are commonly considered apoptosis-related, whereas RIP2, cleaved caspase-3, and cytochrome c are antiapoptosis-related proteins. Our data suggest that proBDNF binds to the p75NTR receptor and activates the RhoA-JNK signaling pathway to promote the expression of apoptosis-related proteins (PSD95, synaptophysin, and P-cofilin) and inhibit the expression of antiapoptosis-related proteins (RIP2, cleaved caspase-3, and cytochrome c).

Postsynaptic density protein 95 (PSD95) is the most important and abundant scaffolding protein of the postsynaptic membrane [42]. It mainly exists in mature excitatory glutamatergic synapses and is the main receptor of the postsynaptic membrane. PSD95 plays an important role in synaptic plasticity. Our data showed that changes in PSD95 levels were induced in the PSD model. The expression of PSD95 was decreased in the PSD model, which was reversed by recombinant p75NTR and siJNK pretreatment.

### p75NTR, JNK1, and RhoA are involved in occurrences of PSD

p75NTR plays a vital biological role in nervous system development [43]. It is mainly expressed during the early development of nerve cells. p75NTR induces cell proliferation, migration, differentiation, survival, apoptosis, synapse establishment, and nerve formation through different signal transduction pathways, and plays multiple biological functions. p75NTR binds to proBDNF and activate downstream signal transduction pathways, such as the RhoA pathway. RhoA is a member of the Rho subfamily of the Rho family in the small G protein superfamily [44]. RhoA, containing eight single loops and six multi-stranded folded fragments, is the main signaling protein that regulates axon regeneration. RhoA has multiple targets [41, 45, 46]. JNK also known as a stress-activated protein kinase, which belongs to the mitogen-activated protein kinase family [19], is one such target [20, 47]. The JNK signaling pathway can be stimulated by a variety of factors outside the cell causing a series of reactions inside the cell. When activated by upstream regulatory factors, JNK translocates to the nucleus and regulates the expression of downstream target genes or the activity of target proteins. In addition to its role in cell proliferation, death, and repair, studies have found that the JNK signaling pathway is related to neurodegeneration, ischemia-reperfusion, and other diseases [21, 48, 49].

In our study, the results showed that the protein level changes in p75NTR, JNK, and RhoA are related to the proBDNF protein level changes induced by PSD. We confirmed that proBDNF, p75NTR, JNK1, and RhoA are decreased after treatment with recombinant p75NTR and siJNK. PSD is closely associated with the upregulation of p75NTR, JNK1, and RhoA in neuronal cells. In addition, the expression of these proteins is decreased after treatment with recombinant p75NTR and siJNK. These data suggest that under PSD-like conditions, proBDNF binds to the p75NTR receptor and activates the RhoA/JNK signaling pathway.

In summary, proBDNF protein expression in neuronal cells was increased in the PSD-like model. proBDNF binds to the p75NTR receptor and activates the RhoA-JNK signal in downstream cells to promote the expression of apoptosis-related proteins (PSD95, synaptophysin, and P-cofilin) to promote cellular apoptosis and inhibit the regeneration of nerve synapses, thereby promoting the pathogenesis of PSD-like processes.
